# Perceived contributions of long-term peer-led cooking activities to community resilience: a case study of nuclear evacuees in Fukushima

**DOI:** 10.3389/fpubh.2026.1784482

**Published:** 2026-09-24

**Authors:** Kanako Sato, Toshiya Kuchii, Ai Kuroda, Yuri Kinoshita, Yoshiharu Fukuda

**Affiliations:** 1 Department of Food and Nutrition, Kanazawa Gakuin College, Kanazawa, Ishikawa, Japan; 2 Japan Foundation for AIDS Prevention, Tokyo, Japan; 3 Teikyo University Graduate School of Public Health, Tokyo, Japan; 4 Department of Health and Nutritional Sciences, Faculty of Health Sciences, Aichi Gakuin University, Nisshin, Aichi, Japan

**Keywords:** community project, cooking class, disaster response, Great East Japan Earthquake, peer-led, social interaction

## Abstract

**Introduction:**

Following the Great East Japan Earthquake, the residents' association of the Shimokajiro housing complex, with support from the Ajinomoto Foundation, implemented cooking classes. This study aimed to examine the significance of these classes after external support ended.

**Methods:**

In March 2024, focus group interviews were conducted with 14 users (11 women and 3 men; mean age, 77.5 years) of a community meeting space in the Fukushima Prefectural Shimokajiro housing complex, which houses evacuees from the nuclear accident. The collected data were qualitatively analyzed using thematic analysis based on the Braun and Clarke method.

**Results:**

Two key benefits were identified regarding the cooking classes conducted by the residents' association. At the individual level, the cooking classes created opportunities for social participation, promoted learning and self-efficacy, and enabled participants to reconnect with aspects of everyday life disrupted by the disaster. At the community level, resident-led activities fostered mutual care and informal monitoring, contributing to the prevention of social isolation and strengthening community cohesion. In the absence of traditional family-based support systems, residents developed weak social ties that enhanced community resilience.

**Conclusion:**

Resident-led cooking activities can provide a sustainable foundation for long-term social recovery in disaster-affected settings. In situations where returning to original communities is not feasible, rebuilding everyday practices through peer-led initiatives may represent a critical pathway for community reconstruction. These findings underscore the importance of resident-led initiatives in fostering social connectedness during disaster recovery.

## 1 Introduction

On March 11, 2011, the Great East Japan Earthquake (hereafter, “the earthquake”) struck, causing a magnitude 9.0 earthquake and subsequent tsunami that left approximately 20,000 people dead or missing (1). Beginning the following day, hydrogen explosions occurred in Units 1 (March 12), 3 (March 14), and 4 (March 15) of the Tokyo Electric Power Company's (TEPCO) Fukushima Daiichi Nuclear Power Plant, releasing radioactive materials (hereafter, “the nuclear accident”). The earthquake and the nuclear accident forced the evacuation of approximately 380,000 people within 1 week. As of February 2024, approximately 30,000 individuals remain unable to return to their homes (1). This disaster was characterized not only by the destruction caused by the earthquake and tsunami but also by the unprecedented scale of the nuclear accident, making it one of the most complex large-scale disasters in modern history. By combining the impacts of natural hazards—the earthquake and tsunami—with those of a technological disaster, the Fukushima disaster created complex governance challenges related to institutional responsibility and infrastructure transformation (2).

Disaster survivors lost family members, friends, and homes and experienced the collapse of local communities because of prolonged evacuation following the nuclear accident. As reconstruction progressed, evacuees underwent multiple transitions—from emergency shelters to temporary housing and eventually to public disaster housing (3). Each relocation resulted in the dissolution of established communities, with social ties repeatedly reconstructed in unfamiliar environments. In addition, families who had lived together before the earthquake were frequently separated. Repeated displacement intensified psychological distress (4) and social isolation, whereas social connectedness has been identified as a key protective factor against these outcomes (5). Prolonged evacuation was also associated with deteriorating dietary habits (6). Relocation to temporary housing reduced household size and limited opportunities for cooking because of restricted kitchen space and other environmental constraints. As a result, fewer residents prepared their own meals, relying increasingly on prepackaged side dishes, boxed lunches, instant meals, and frozen foods (7). Previous studies have demonstrated an association between psychological distress among evacuees and reduced intake of specific food groups (8). Furthermore, low dietary diversity has been observed among older adults residing in public disaster housing, underscoring the need for nutritional support (9).

In October 2011, the Ajinomoto Group (10–12) launched the Red Apron Project to improve the deteriorating dietary habits and nutritional status of disaster survivors while also revitalizing local communities through food-based interventions (13). The project was subsequently transferred to the Ajinomoto Foundation (TAF). Unlike conventional cooking classes, this initiative aimed to rebuild social connections among disaster survivors by encouraging participants to cook and eat together. Under the theme “cooking together and eating together,” the project introduced outreach-style cooking classes in collaboration with partner organizations, including government agencies, Councils of Social Welfare, private organizations, neighborhood associations, and universities operating in disaster-affected areas. Over 8.5 years, until March 2020, these classes were conducted 3,771 times across 51 cities, towns, and villages in the three most affected prefectures—Iwate, Miyagi, and Fukushima—reaching 54,434 participants (Supplementary Figure 1) (14). Participation in cooking programs has been shown to improve cooking confidence (15, 16), enhance self-efficacy and knowledge (17), and strengthen social connectedness (18, 19). Although direct support concluded in March 2020, logistical assistance has continued, including the provision of cooking utensils, such as gas stoves, pans, bowls, and recipes, to communities and organizations intending to sustain the classes independently (20). Since 2016, training workshops have facilitated the implementation of cooking classes while encouraging a transition from passive recipients of support to active residents who initiate and participate autonomously in community activities.

The project's participatory cooking classes are thought to improve the quality of life of disaster survivors while fostering social cohesion. An independent evaluation described the initiative as “an innovative intervention model that ‘energizes people's minds and bodies”' (21). Moreover, the initiative has been reported to contribute to enhanced community resilience (22). Implementation in Fukushima—where the earthquake, tsunami, and nuclear accident compounded the disaster—further underscores the significance of this initiative (23).

Accordingly, this study examines sustained peer-led activities among evacuees residing in Shimokajiro Danchi, Fukushima Prefecture. In disaster-affected settings, such activities extend beyond mutual support by helping survivors restore daily routines and community functioning. Castellano (24) reported that peer-led activities are associated with greater resilience among disaster survivors experiencing adversity and trauma. Putera et al. (25) highlighted the effectiveness of a hybrid model that combines public institutions with resident-led activities. Although previous initiatives have included social gatherings and exercise classes, research on long-term cooking classes remains limited. This study examines the significance of cooking classes as a resident-led peer activity for individual disaster survivors and the broader disaster-affected community. A key strength of this study is its focus on long-term, food-based initiatives led by disaster-affected residents in the post-disaster context.

## 2 Materials and methods

This study employed a qualitative case study design based on focus group interviews. The focus group interviews were conducted by members of the research team who were neither affiliated with the participants' organizations nor involved in their activities. Participants were residents who used the community meeting space at Shimokajiro Danchi, a public disaster-recovery housing complex operated by Fukushima Prefecture for individuals forced to evacuate following the nuclear accident. The interviews were conducted in March 2024 in the community meeting space at Shimokajiro Danchi, Iwaki City, Fukushima Prefecture. Officers of the residents‘ association invited users of the community meeting space to participate. No residents declined participation. Ultimately, 14 individuals agreed to participate and provided written informed consent. The interviews were conducted in three groups, organized to ensure that participants felt comfortable speaking with one another.

Participants included 11 women and 3 men, with a mean age of 77.5 years. Eleven were evacuees from Namie, Okuma, and Tomioka, whereas three resided in Iwaki City (Table 1). Interviews were conducted using a semi-structured interview guide and lasted approximately 60–90 min per group. The moderators were members of the research team: TK, the second author, moderated Group 1; KS, the first author, moderated Group 2; and YK, the fourth author, moderated Group 3. All interviews were conducted in Japanese and audio-recorded using an IC recorder.

**Table 1:** Attributes and characteristics of interviewees.

Interview group	Participant No.	Age (years)	Sex	Person living together	Pre-disaster residential area	Role in the community	Participation in cooking classes^※^	Role in cooking classes
1	1	69	Male	Spouse	Namie-machi	Neighborhood association staff	From program initiation	Preparation Treasurer
	2	76	Male	Spouse	Okuma-machi	Not reported	Since 2016 (80 sessions)	Preparation Instructor
	3	71	Female	None	Okuma-machi	Not reported	Since 2016 (80 sessions)	Preparation
	4	65	Female	Spouse	Namie-machi	Not reported	Since 2023 (13 sessions)	Preparation
	5	69	Female	None	Namie-machi	Neighborhood association staff	Since 2016 (80 sessions)	Preparation
2	6	93	Female	None	Namie-machi	Not reported	Since relocation to temporary housing	Not reported
	7	70	Female	Spouse	Namie-machi	Not reported	Since relocation to temporary housing	Treasurer
	8	85	Female	Spouse, child, grandchild	Iwaki-shi	Not reported	Since 2022 (5 sessions)	Not reported
	9	81	Female	Spouse, child	Iwaki-shi	Not reported	Since 2022 (5 sessions)	Not reported
	10	81	Female	Spouse	Namie-machi	Not reported	Since 2015 (10 sessions)	Not reported
3	11	88	Female	Spouse, child	Iwaki-shi	Not reported	10 sessions	Not reported
	12	82	Female	None	Tomioka-machi	Not reported	From program initiation	Not reported
	13	71	Female	Spouse	Okuma-machi	Not reported	3 sessions (discontinued)	Not reported
	14	84	Male	Spouse, child	Tomioka-machi	Not reported	No participation	Not reported

^※^ Participation history includes classes attended at prior evacuation sites (such as temporary housing) or other venues before the program officially began at Shimokajiro Danchi in 2017.

Interview data were analyzed using the six-step approach of Braun and Clarke's thematic analysis (26). The research team prepared verbatim transcripts from the audio recordings. Notable features were systematically identified across the dataset to generate initial codes, which were then organized and integrated into themes. Candidate themes were reviewed against the entire dataset to ensure that they accurately reflected the data and to enhance analytic rigor. The essence of each theme was identified, and each was assigned a concise, descriptive name that reflected its analytic content. Microsoft Excel was used throughout the coding, classification, and organization process.

To improve the reliability of the analysis, four co-authors independently coded the data, and any disagreements were discussed until consensus was reached. The themes, codes, and representative quotations were then carefully translated from Japanese into English, with close attention to preserving the original meanings and nuances. The research team remained mindful of how their professional backgrounds and values might influence data interpretation, engaging in reflexivity through analytic memos and team discussions. The analysis was conducted by the research team independently of the funder. The funder was not involved in the study design, data management, analysis, interpretation of the findings, or the decision to submit the manuscript for publication.

This study was conducted in accordance with the Declaration of Helsinki. It was approved by the ethics committee of Tohoku Seikatsu Bunka University and Junior College (approval no. R5-20). Written informed consent was obtained from all participants. In addition, written informed consent was obtained for the publication of any potentially identifiable images or data included in this article. The study was funded by TAF. However, the researchers explained to the participants that the study was conducted independently of the funding organization and that they could freely express positive or negative opinions, including evaluations of the funding organization, without risk of disadvantage.

## 3 Results

### 3.1 Overview of Shimokajiro Danchi, Fukushima Prefectural housing

Currently, Fukushima Prefecture maintains 71 public disaster-recovery housing complexes across 15 municipalities, comprising 61 prefecturally operated and 10 municipally operated complexes (27). These housing complexes were developed to accommodate individuals and families forced to evacuate following the nuclear accident triggered by the Great East Japan Earthquake. Shimokajiro Danchi is one such housing complex, located in Iwaki City, approximately 50 km from the Fukushima Daiichi Nuclear Power Plant (Figure 1). Due to this geographical separation, residents have had limited access to administrative services in their original municipalities. The complex consists of six five-story reinforced concrete apartment buildings, totaling 200 housing units (28). In February 2015, evacuees from Namie, Futaba, Okuma, and Tomioka began relocating to the complex.

**Figure 1:**
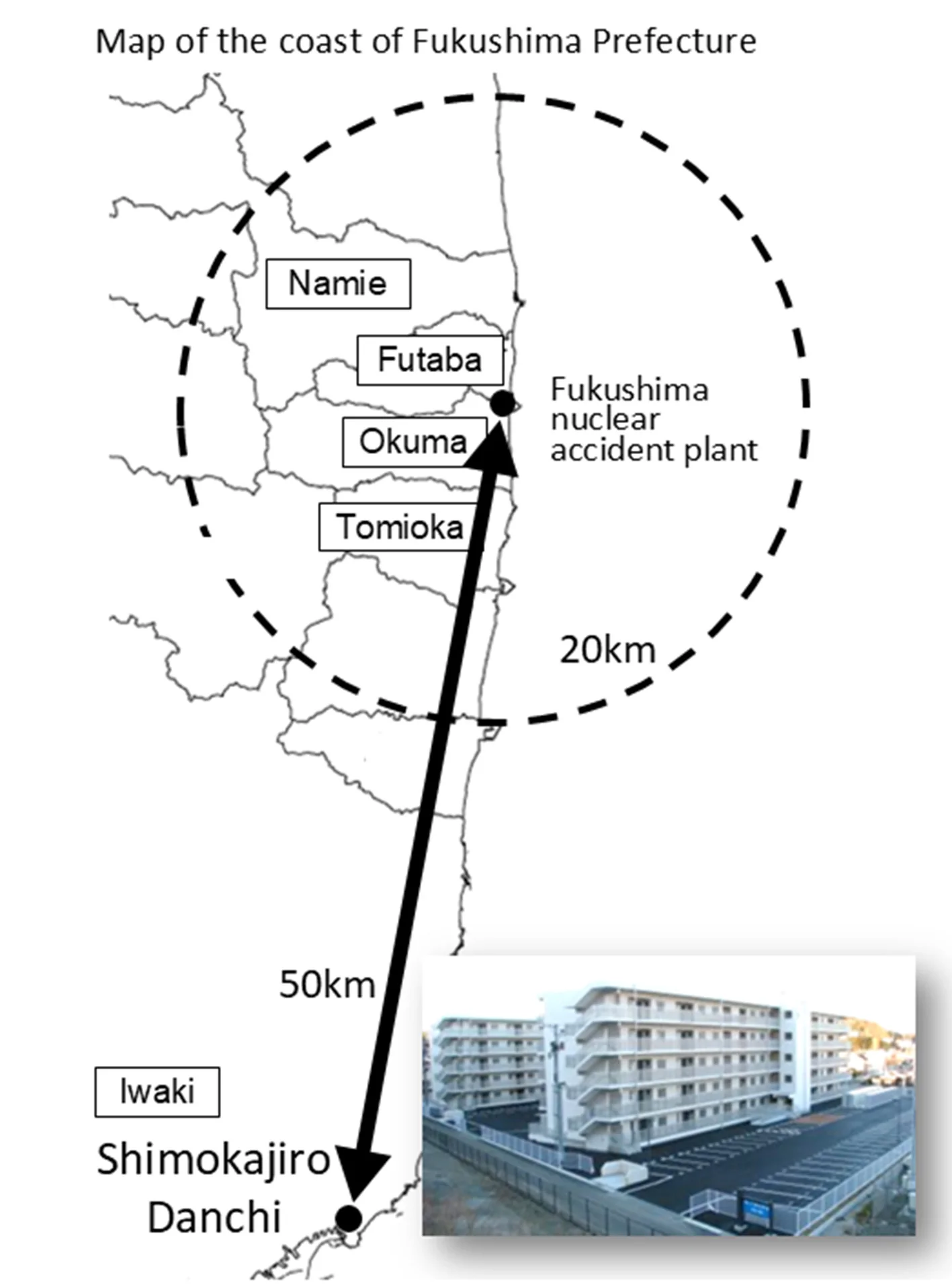
Locations of the towns of Namie-machi, Futaba-machi, Okuma-machi, and Tomioka. Photograph of the Shimokajiro Danchi buildings. Reproduced with official permission from the Reconstruction and Comprehensive Planning Division, Fukushima Prefectural Government (28). Source: https://www.pref.fukushima.lg.jp/site/portal/ps-fukkoukoueigenba008.html.

The day after the earthquake, when the nuclear accident occurred, the evacuation zone expanded from a 3-km radius to 10 km and then to 20 km. Residents evacuated under conditions of considerable uncertainty, and many had little time to gather their belongings. All residents of Futaba-machi, Okuma-machi, and Tomioka-machi were evacuated because their towns were located within the 20-km radius. Although part of Namie-machi lay outside this zone, it was later designated a planned evacuation area, prompting the evacuation of all its residents. As reconstruction progressed, evacuation sites changed repeatedly—from emergency shelters to temporary housing and eventually to public disaster housing.

In 2017, 6 years after the earthquake and nuclear accident, evacuation orders for parts of Namie-machi and Tomioka-machi were partially lifted. This was followed by similar changes in Okuma-machi in 2019 and Futaba-machi in 2020 under designations such as “zones in preparation for the lifting of evacuation orders” and areas with “lifting of residential restrictions” (Table 2). Although the populations of these towns have gradually increased, they remain far below their pre-earthquake levels. Table 3 compares the pre-earthquake and current populations.

**Table 2:** Evacuation routes from Namie-machi, Futaba-machi, Okuma-machi, and Tomioka-machi.

Namie-machi	March 12, 2011	Evacuation order issued within a 20-km radius; evacuation to the Tsushima area of the same town^※1^
	March 15, 2011	Evacuation to Nihonmatsu-shi^※1^
	March 31, 2017	Evacuation orders lifted in areas prepared for lifting and in residency restriction areas^※2^
Futaba-machi	March 12, 2011	All residents ordered to evacuate; evacuation to Kawamata-machi^※1^
	March 19, 2011	Evacuation to Saitama Super Arena^※1^
	March 4, 2020	Evacuation orders lifted in areas prepared for lifting and in residency restriction areas^※2^
Okuma-machi	March 12, 2011	All residents ordered to evacuate; evacuation to Tamura-shi, Koriyama-shi, Mihara-machi, and Ono-machi^※1^
	March 19, 2011	Evacuation to Aizuwakamatsu-shi^※1^
	April 10, 2019	Evacuation orders lifted in areas prepared for lifting and in residency restriction areas^※2^
Tomioka-machi	March 12, 2011	All residents ordered to evacuate; 6,000 residents evacuated to Kawauchi-mura^※1^
	March 16, 2011	Evacuation to Big Palette Fukushima^※1^
	April 1, 2017	Evacuation orders lifted in areas prepared for lifting and in residency restriction areas^※2^
Shimokajiro Danchi	February 2015	Move-in began
	October 2017–February 2020	Cooking classes held with support from TAF
	March 2020	Cooking classes independently organized by the residents' association
	March 2024	Focus group interviews conducted

^※1^ Partially excerpted from the report of the TEPCO Fukushima Nuclear Power Plant Accident Investigation Committee.

^※2^ Partially excerpted from the Fukushima Reconstruction Portal Site section on evacuation zone establishment (https://www.pref.fukushima.lg.jp/uploaded/attachment/687759.pdf).

**Table 3:** Population changes in the towns of Namie, Futaba, Okuma, and Tomioka.

Town	Population at the time of the earthquake (As of March 11, 2011)	Current residential and evacuation status of residents				
		Current population	Outside Fukushima Prefecture	Inside Fukushima Prefecture	Inside town	Outside town
Namie	21,434^※1^	19,049^※2^	5,902^※2^ (31.0%)	13,147^※2^ (69.0%)	1,442^※2^ (7.6%)	11,705^※2^ (61.4%)
		As of September 30, 2024	As of September 30, 2024	As of September 30, 2024	As of September 30, 2024	As of September 30, 2024
Futaba	7,147^※1^	6,457^※3^	2,667^※3^ (41.3%)	3,790^※3^ (58.7%)	68^※3^ (1.1%)	3,722^※3^ (57.6%)
		As of September 30, 2024	As of September 30, 2024	As of September 30, 2024	As of September 30, 2024	As of September 30, 2024
Okuma	11,505^※1^	9,961^※4^	2,154^※4^ (21.6%)	7,807^※4^ (78.4%)	848^※4^ (8.5%)	6,959^※4^ (69.9%)
		As of September 30, 2024	As of September 30, 2024	As of September 30, 2024	As of September 30, 2024	As of September 30, 2024
Tomioka	15,960^※1^	11,368^※5^	1,647^※5^ (14.5%)	9,721^※5^ (85.5%)	2,540^※5^ (22.3%)	7,181^※5^ (63.2%)
		As of October 1, 2024	As of October 1, 2024	As of October 1, 2024	As of October 1, 2024	As of October 1, 2024

^※1^ Fukushima Prefecture Evacuation Area Wide-Area Public Transportation Network Formation Plan (2018). https://www.pref.fukushima.lg.jp/uploaded/attachment/547406.pdf (Accessed December 20, 2025).

^※2^ Those who had a certificate of residence in Namie-machi on March 11, 2011. Namie Town Hall. Housing and Evacuation Status (2024). https://www.town.namie.fukushima.jp/soshiki/3/37332.html (Accessed December 20, 2025).

^※3^ Population after subtracting deaths from the population at the time of the disaster and including new residents of Futaba-machi and newborns. Excludes new residents who moved to Futaba-machi after the evacuation order was lifted for the Specified Reconstruction and Revitalization Base Area on August 30, 2022. Futaba Town Hall. Housing and Evacuation Status (2024). https://www.town.fukushima-futaba.lg.jp/11016.htm (Accessed December 20, 2025).

^※4^ Currently registered residents of Okuma-machi. Okuma Town Hall. Housing and Evacuation Status (2024). https://www.town.okuma.fukushima.jp/soshiki/juminzeimu/29031.html (Accessed December 20, 2025).

^※5^ Currently registered residents of Tomioka-machi. Tomioka Town Hall. Housing and Evacuation Status (2024). https://www.tomioka-town.jp/soshiki/jumin/6213.html (Accessed December 20, 2025).

### 3.2 Peer-led activities in Shimokajiro Danchi

In Japan, approximately 300,000 residents‘ associations operate nationwide. These organizations are established based on local community ties and are voluntarily managed by residents (29, 30). Residents' associations engage in community-based collective activities that support the maintenance and development of healthy local communities. Such activities include facilitating communication among residents, improving the local environment, and managing shared community facilities (29). Initially, each town maintained its own neighborhood association to support residents from the same hometown. However, to address concerns such as social isolation among older adults, community revitalization, and the need for intergroup collaboration, the associations were merged in 2016, 1 year after relocation. Although the unified neighborhood association hosted cafés and other community gatherings, there remained a need for more interactive and participatory activities. In 2017, TAF proposed initiating a cooking class through the Council of Social Welfare and the local non-governmental organization, Minpuku (31).

Viewing it as a valuable opportunity to acquire cooking skills and promote social engagement, the residents‘ association launched the initiative. Over the following 3 years, until February 2020, a total of 13 cooking classes were conducted, engaging 228 participants. With operational support from TAF, the residents' association continued the program as a peer-led initiative. In March 2020, activities were temporarily suspended because of the COVID-19 pandemic. After evaluating the risks of social isolation and infection and consulting with the prefectural administrator, the association decided to resume community programs, including cooking classes. Currently, cooking classes are held independently every 3 months (Supplementary Figure 2). In addition to cooking classes, the neighborhood association organizes a variety of events throughout the year, including cafés and karaoke sessions (twice weekly), shamisen lessons and fitness drumming (weekly), and seasonal celebrations, such as a summer party and a Christmas party (Supplementary Figure 3). As of February 2024, 9 years after its establishment, Shimokajiro Danchi comprised 101 evacuee households, of which approximately 50% were single-person households, and approximately 70% of residents were aged ≥65 years.

### 3.3 Results of focus group interviews

The interview data were analyzed under three themes: (i) reported benefits at the individual level, (ii) reported benefits at the community level, and (iii) reasons for residents' participation or non-participation in the cooking classes. The identified themes and corresponding codes, together with representative quotations, are presented in Table 4.

**Table 4:** Themes, codes, and representative quotations from focus group interviews.

Theme	Code	Quotation
Reported benefits: individual level	Creating opportunities to go out	It's fun, it's fun! More than anything, it's about communicating with people and enjoying each other's presence. Since I live alone, I want to come to a place like this and chat.
		I participate in the classes because it's fun to cook and eat together.
	Learning and personal growth	I get to make dishes I've never tried at home before, and I realize how good they taste when I do.
		It's different from cooking alone, and Ajinomoto's menus are easy to follow. The recipes use familiar ingredients and are simple to prepare.
		I can cook the dishes myself, it's easy. That's why I'm happy.
Reported benefits: community level	Prevention of lonely deaths and social isolation within the community	I think it's great for residents to gather because it allows everyone to check in on each other.
		If someone notices, “Hey, this person isn't here today. What's going on?”, they might find out, “Oh, they have a doctor's appointment today,” and so on.
	Strengthening cohesion in local communities	I have made more friends through the cooking class. I'm not a very active person, so I'm grateful for the opportunity to participate and connect with others.
		I believe cooking classes strengthen bonds and encourage cooperation. I feel that people are connecting when someone reaches out and others come together to support them.
		The organizers of this class are leading it very effectively. After all, events like Nagashi Somen (Flowing Noodles) in the summer wouldn't be possible without the efforts of those who work hard to make them happen. In the end, whether it's a coffee gathering or another community event, these spaces bring people together and foster a sense of connection.
Reasons for residents participating or not participating in the cooking classes	Reasons for participating	The flyers were what got me interested.
		To recruit participants, we start by distributing flyers to all the households.
		I knew someone who participated, and they invited me, saying, “Why don't you join?”
		The food is easy to make and nutritious, that's why I like it.
		The food tastes delicious.
	Reasons for not participating	I've never participated in the classes. It's not that I'm not interested—it's just that I don't have the time. The schedule often conflicts with my doctor's appointments or work.
		If the class happens to coincide with another event in Tomioka-machi, some people won't be able to attend.
		I have trouble in my eyesight due to the aftereffects from a brain surgery 15 years ago. So, if I try to cook at the same pace as everyone else, I get exhausted. That's why I don't participate.
	Thoughts of participants	We can't force people to participate, even if we reach out to them.
		Those who are isolated may need a different form of connection.
		Still, I truly hope that non-participants will join in, so they don't become isolated.

#### 3.3.1 Reported benefits: individual level

##### 3.3.1.1 Creating opportunities to go out

During the cooking classes, participants prepared meals together and naturally engaged in conversation. This shared activity provided a meaningful opportunity for social interaction amid the isolation of post-disaster life, strengthening interpersonal relationships. The enjoyable atmosphere also motivated participants to continue attending, thereby increasing their social engagement and opportunities to leave their homes, as reflected in the following comments:

“*It's fun, it's fun! More than anything, it's about communicating with people and enjoying each other's presence. Since I live alone, I want to come to a place like this and chat.”*

“*I participate in the classes because it's fun to cook and eat together.”*

##### 3.3.1.2 Learning and personal growth

The menus used in the cooking classes incorporated simple, familiar ingredients, making them suitable for the limited kitchen facilities available in temporary housing. The meals were also flavorful and nutritionally balanced, appealing to participants who may have experienced a loss of appetite following the disaster. Many of the dishes were unfamiliar to the participants, providing opportunities for learning and personal growth.

“*I get to make dishes I've never tried at home before, and I realize how good they taste when I do.”*

“*It's different from cooking alone, and Ajinomoto's menus are easy to follow. The recipes use familiar ingredients and are simple to prepare.”*

“*I can cook the dishes myself, it's easy. That's why I'm happy.”*

#### 3.3.2 Reported benefits: community level

##### 3.3.2.1 Prevention of lonely deaths and social isolation within the community

Given that many older adults live alone in the apartment complex, loneliness and social isolation present significant challenges. In this context, events organized by the neighborhood association—including cooking classes—encouraged residents to monitor one another's wellbeing. These gatherings played a key role in fostering mutual awareness and support, helping prevent social isolation and lonely deaths.

“*I think it's great for residents to gather because it allows everyone to check in on each other.”*

“*If someone notices, ‘Hey, this person isn't here today. What's going on?', they might find out, ‘Oh, they have a doctor's appointment today,' and so on.”*

##### 3.3.2.2 Strengthening cohesion in local communities

The cooking classes served as more than culinary activities; they became a venue for reconnecting residents who had been separated by the disaster. Residents were actively involved in organizing the events, fostering a shared sense of purpose and reinforcing community cooperation. The classes were held regularly, creating consistent opportunities for interaction and helping to strengthen community ties. Respondents noted that the sustainability of these efforts depended not only on the leadership of neighborhood association officials but also on mutual communication and collaboration among residents. They emphasized the importance of shared spaces and meaningful interpersonal connections in rebuilding community cohesion.

“*I have made more friends through the cooking class. I'm not a very active person, so I'm grateful for the opportunity to participate and connect with others.”*

“*I believe cooking classes strengthen bonds and encourage cooperation. I feel that people are connecting when someone reaches out and others come together to support them.”*

“*The organizers of this class are leading it very effectively. After all, events like Nagashi Somen (Flowing Noodles) in the summer wouldn't be possible without the efforts of those who work hard to make them happen. In the end, whether it's a coffee gathering or another community event, these spaces bring people together and foster a sense of connection.”*

#### 3.3.3 Reasons for participation or non-participation in the cooking classes

##### 3.3.3.1 Reasons for participating

Flyers distributed to all households played a significant role in attracting participants, both directly and through word of mouth. Positive experiences during the cooking classes also encouraged many participants to return for future sessions.

“*The flyers were what got me interested.”*

“*To recruit participants, we start by distributing flyers to all the households.”*

“*I knew someone who participated, and they invited me, saying, ‘Why don't you join?”'*

“*The food is easy to make and nutritious, that's why I like it.”*

“*The food tastes delicious.”*

##### 3.3.3.2 Reasons for not participating

Three main factors were identified as barriers to participation: (i) scheduling conflicts, such as overlapping appointments or community events; (ii) health concerns, including physical limitations; and (iii) psychological barriers, such as discomfort with unfamiliar settings. A substantial proportion of residents cited health-related issues, highlighting the importance of reducing both physical and emotional barriers to encourage broader participation.

“*I've never participated in the classes. It's not that I'm not interested—it's just that I don't have the time. The schedule often conflicts with my doctor's appointments or work.”*

“*If the class happens to coincide with another event in Tomioka-machi, some people won't be able to attend.”*

“*I have trouble in my eyesight due to the aftereffects from a brain surgery 15 years ago. So, if I try to cook at the same pace as everyone else, I get exhausted. That's why I don't participate.”*

##### 3.3.3.3 Thoughts of participants

Participants expressed a strong desire to include residents who had not yet joined the cooking classes, hoping that they could also experience the social benefits of participation. Rather than pressuring them, participants preferred to provide ongoing encouragement. These efforts fostered a sense of mutual support within the community, promoting both individual initiative and collective care among residents.

“*We can't force people to participate, even if we reach out to them.”*

“*Those who are isolated may need a different form of connection.”*

“*Still, I truly hope that non-participants will join in, so they don't become isolated.”*

## 4 Discussion

### 4.1 Participant-perceived contributions

This study identified three participant-perceived contributions at the individual level, indicating that the cooking classes served purposes beyond providing food to disaster-affected residents.

First, these activities served as a meaningful source of enjoyment in post-disaster life, which might otherwise become monotonous, and provided opportunities for more frequent outings. Daily routines centered on preparing and consuming meals alone were prone to repetition; in contrast, participation in the cooking classes introduced variety into everyday life and fostered intrinsic motivation to “want to go again.” Tashiro et al. (18) similarly reported that participation in cooking classes increased opportunities to go out.

Second, the cooking classes provided a setting for learning and personal growth. The menus consisted of simple dishes prepared with readily available ingredients that could be replicated even in the limited cooking environments of temporary and disaster-recovery housing. Through successful cooking experiences and learning new dishes, participants' cooking confidence increased, which in turn contributed to enhanced self-efficacy. Previous studies have reported that participation in cooking classes contributes not only to improved nutritional status but also to enhanced self-efficacy and strengthened social connectedness (15–17). The findings of the present study are consistent with these reports.

Third, cooking activities, as an extension of everyday life, provided disaster survivors with an opportunity to reconnect with aspects of ordinary life that had been disrupted by the disaster. For residents who had lost their homes and shared family lives because of the earthquake and nuclear accident, the experiences described as “delicious” and “enjoyable” during the cooking classes helped them reconnect with meaningful aspects of their former daily lives. Previous research suggests that engagement in everyday practices holds social significance and provides psychosocial benefits (32).

### 4.2 Value of peer-led activity

Since 2017, the cooking classes at Shimokajiro Danchi have been managed by the neighborhood association in collaboration with TAF and have been fully self-organized by the association since 2020. These voluntary, peer-led activities promoted resident autonomy. Residents perceived that, rather than merely participating in the cooking classes, taking on roles themselves and helping sustain the activities deepened relationships among residents and strengthened social cohesion. This involvement may have fostered an environment in which residents naturally looked out for one another, such as noticing when someone was absent or expressing concern about a neighbor's health condition.

Previous studies have reported that peer-led activities help maintain and promote independence (33) and enhance the resilience of disaster survivors (24), findings that are consistent with those of the present study. The resident-led voluntary activities in this case also provide a concrete example of Putera et al.'s (25) hybrid model of disaster resilience, which emphasizes “adaptive institutions, community participation, and the integration of local knowledge and technology”. The cooking activities at the Shimokajiro housing complex can be viewed as a part of a “broader community resilience ecosystem” that extends beyond a single program to support community-wide mutual care and social bonds. These findings suggest that disaster recovery efforts should extend beyond external support to include mechanisms that enable and sustain resident-led voluntary activities.

### 4.3 Strengthening connections and building community

A perceived contribution identified by residents was that taking an active role in organizing and running the cooking classes fostered “connectedness” and “cooperation,” deepening trust and solidarity and thereby contributing to enhanced community resilience.

Many residents were older adults living alone who had lost family members, friends, and homes and continued to live in evacuation without the possibility of returning to their hometowns. In rural Japan, communities have traditionally been structured around the IE system, a social framework in which family members support one another (34). However, the unprecedented disaster disrupted these family ties and long-standing social bonds. In the absence of such established familial relationships, residents developed weak but meaningful social ties with others in the community through various activities, including cooking classes. In a housing complex with a high proportion of older adults living alone, such peer connections played an important role in preventing social isolation and reducing the risk of lonely deaths.

Furthermore, residents adapted to the drastically altered circumstances brought about by the disaster and reconstructed a new community. This process may support the interpretation that “resilience” involves not merely a pre-disaster state but rather a shift toward transformative resilience, in which adaptation and recovery occur within a newly established social context. Goniewicz et al. (35) reported that health governance in the European Union following the COVID-19 pandemic shifted toward a form of “transformative resilience,” characterized by responses that extend beyond simple crisis management. Because this approach emphasizes adaptation to new conditions rather than restoration of previous states, it is consistent with the findings of the present study.

### 4.4 Reasons for non-participation and mutual care among residents

This study identified several barriers to participation among residents who did not attend the cooking classes. The first involved physical and health-related constraints: some residents cited visual impairment resulting from the aftereffects of brain surgery or fatigue from trying to keep pace with others. These observations suggest that even when residents are motivated to participate, physical limitations may become psychological barriers to social participation. The second barrier was scheduling conflicts, including medical appointments and other community events. Conflicts with events in residents' hometowns may represent a constraint unique to life as an evacuee, reflecting their efforts to maintain ties with their original communities.

At the same time, participating residents wanted non-participants to benefit from the social connections fostered through the cooking classes. Although participation was voluntary, they continued to invite and encourage non-participants to join. Through these efforts, an informal culture of mutual care developed, regardless of whether residents directly participated in the classes. The activity therefore served not only as an opportunity to cook together but also as an important foundation for strengthening self-help and mutual aid within the community.

This study has two limitations. First, it is based on the unique context of a nuclear disaster. Resilience in disaster recovery is generally defined as recovery to the pre-disaster state; however, in the present study, returning to residents' hometowns was virtually impossible. Accordingly, the conventional concept of resilience cannot be directly applied to this setting. Nevertheless, a key strength of this study lies in its focus on peer-led activities under conditions in which return was not possible. These findings illustrate transformative resilience, whereby social ties among local residents—particularly weak social ties—contributed to the formation of a new community.

Second, participants were limited to residents who agreed to participate after being invited by officers of the residents' association at Shimokajiro Danchi, specifically those who used the community meeting space. This sampling approach excluded residents who did not use the meeting space and may therefore have introduced selection bias.

Future research should examine similar peer-led activities across a wider range of disaster contexts and community settings.

This study contributes to understanding how everyday practices function as mechanisms of long-term social recovery in Shimokajiro Danchi, Fukushima Prefecture. Through cooking classes, residents reconstructed aspects of their ordinary daily lives, enhanced their self-efficacy, and strengthened their social connectedness. Moreover, the transition to resident-led peer activities fostered an environment in which residents looked out for one another, established a social foundation for preventing isolation, and contributed to community resilience. In disaster recovery, resident-led activities that foster “connectedness” may offer valuable insights for similar contexts.

## Data Availability

The datasets presented in this article are not readily available because they contain sensitive information related to disaster survivors. Requests to access the datasets should be directed to Kanako Sato, k-sato@kanazawa-gu.ac.jp.
