# Supplementary material for: Perceived contributions of long-term peer-led cooking activities to community resilience: a case study of nuclear evacuees in Fukushima
Source: Front Public Health. 2026 Sep 24;14:1784482. doi: 10.3389/fpubh.2026.1784482 (PMC13647991; doi:10.3389/fpubh.2026.1784482)
Supplement: Supplementary file 1 [file Supplementary_file_1.docx]

**Supplementary Figure 1**　“Red Apron Project” for the Great East Japan Earthquake Reconstruction Support Map of municipalities where we held seminars between October 2011 and February 2020 (51 municipalities in Iwate, Miyagi, and Fukushima). TAF had three bases (red dots) and continued its cooking class activities in 51 locations (blue dots). This map was created by TAF.

**Supplementary Figure 2**　During the cooking class, participants wore aprons, triangular head coverings, and masks, and prepared meals under hygienic conditions (left). After the class, everyone enjoys the meal in a relaxed atmosphere (right). The activities of Shimokajiro Danchi were photographed by Y. Kinoshita.

**Supplementary Figure 3**　Monthly schedule of neighborhood association events posted on the wall of the assembly hall (left). This was provided by Shimokajiro Danchi neighborhood association. In the summer, a bamboo ramp is built in the garden of the assembly hall, where people enjoy Somen in summer party (right). This was photographed by Y. Kinoshita.
